# Growth patterns in early childhood: Better trajectories in Afro-Ecuadorians independent of sex and socioeconomic factors

**DOI:** 10.1016/j.nutres.2017.06.003

**Published:** 2017-08

**Authors:** Sheila Maria Alvim Matos, Leila D. Amorim, Ana Clara P. Campos, Mauricio L. Barreto, Laura C. Rodrigues, Yadira A. Morejón, Martha E. Chico, Philip J. Cooper

**Affiliations:** a Instituto de Saúde Coletiva, Universidade Federal da Bahia, Rua Basílio da Gama, S/N, Campus Universitário Canela, Salvador, 40.110-040, Brazil; b Instituto de Matemática, Universidade Federal da Bahia, Salvador, Bahia; c Infectious Diseases Epidemiology, London School of Hygiene and Tropical Medicine, Keppel Street, London, WC1E 7HT, UK; d Facultad de Ciencias Medicas, de la Salud y la Vida, Universidad Internacional del Ecuador, Quito, Ecuador; e Fundación Ecuatoriana para investigación en Salud, Gaspar de Villaroel E8-25 y Seymour, Quito, Ecuador; f Institute of Infection and Immunity, St George's University of London, Cranmer Terrace, Tooting, London SW17 ORE, United Kingdom

**Keywords:** Growth, Trajectories, Childhood, Ethnicity, Multilevel Models, ECUAVIDA, **E**studio e**CUA**toriano del impacto de infecciones sobre **V**acunas, **I**nmunidad y el **D**esarollo de enfermedades **A**lergicas, HPAB, Hospital “Padre Alberto Buffoni”, WAZ, Weight for Age Z-Score, HAZ, Height for Age Z-Score, WHZ, Weight/Height for Age Z-Score, WHO, World Health Organization, AIC, Akaike Information Criterion

## Abstract

The first years of life are the most dynamic period for childhood growth. There are limited data available on growth patterns of infants and children living in rural Latin America. The aim of this study was to describe the growth patterns from birth to 5 years in children living in a rural District of tropical coastal Ecuador using data from a birth cohort of 2404 neonates. We hypothesize that there would be growth differences according to ethnicity and sex. Evaluations were conducted at birth or until 2 weeks of age and at 7, 13, 24, 36 and 60 months during clinic and home visits. Individual growth trajectories for weight-for-age, height-for-age and weight/height-for-age Z-scores were estimated using multilevel models. Girls were lighter and shorter than boys at birth. However, Afro-Ecuadorian children (versus mestizo or indigenous) were longer/taller and heavier throughout the first 5 years of life and had greater mean trajectories for HAZ and WAZ independent of sex and socioeconomic factors. Our data indicate that ethnicity is a determinant of growth trajectories during the first 5 years of life independent of socioeconomic factors in a birth cohort conducted in a rural region of Latin America.

## 1 Introduction

The most dynamic stage of growth occurs during the first years of life. This period of growth is an important determinant of future health but is vulnerable to social and environmental injuries [1]. Different anthropometric growth patterns observed in children worldwide have been attributed to differences in social and economic development. Linear growth patterns reflect the level of a country's development [2]: populations tend to be taller in high compared to low-income countries. Although genetics influence linear growth, adequate nutrition, living conditions, and development level all contribute to whether an individual achieves his or her maximal stature [2]. Studies from Brazil also have shown that low income, absence of prenatal care and low birth weight were determinants of deficits in weight-for age and height-for-age [3], [4].

Patterns of growth in the uterine environment may determine future health and wellbeing. Rapid weight gain during the first year of life is considered to be a risk factor for obesity and chronic diseases in childhood [5] and adulthood [6], and has been put forward as evidence in support of the fetal origins of disease hypothesis (or the Barker Hypothesis). This suggests that conditions during fetal life and early childhood may increase the risk of chronic diseases in adults [6], [7]. There is also evidence that infants with low birth-weight, whose postnatal catch-up growth is rapid, are more likely to become overweight or obese children [5], [8]. To our knowledge, there are no previously published studies from Ecuador on weight gain in early childhood apart from a study of the effects of micronutrient supplementation on growth during the second year of life [9].

The limited available data on growth patterns of Ecuadorians infants and children are from small cross-sectional studies comparing children between the Andes and Coast [10], [11]. A recent national survey of Ecuadorian infants and children [12] showed high rates of linear growth restriction (25.3%), underweight (6.4%) and anemia (62%) in children less than 12 months of age, although the findings showed a substantial improvement compared to previous surveys [13], [14].

The nutritional transition in Latin America from more traditional to more Western diets has been accompanied by a decline in malnutrition, stunting and underweight, in all age groups and an increase in overweight and obesity. Nutritional interventions deserve greater attention as a means to prevent and treat chronic diseases even in countries where malnutrition was previously extremely common [13].

Stunting is a prevalent problem in Latin American and more frequent among children of low socioeconomic status [12], but there are few data on the effects of ethnicity and sex on childhood growth. In the present study, we investigated the characteristics of growth within a birth cohort and hypothesized that ethnicity and sex would be important determinants of growth patterns during the first 5 years of life in children living in a District of a tropical region of rural Ecuador.

## 2 Methods and materials

### 2.1 Study design

The study was a birth cohort, the ECUAVIDA cohort, of 2404 neonates recruited at the Hospital “Padre Alberto Buffoni” (HPAB) over the period November 2005 to December 2009, in the District of Quinindé, Esmeraldas Province in Northern coastal Ecuador. The main objective of the ECUAVIDA cohort was to investigate the potential effects of intrauterine and postnatal exposures to geohelminth parasites on vaccine immune responses in infancy, and the development of allergic sensitization and allergic inflammatory diseases in childhood. The methodology of the cohort study, including power and sample size estimations for primary outcomes, has been described previously in detail [15].

### 2.2 Study population and area

Quinindé is a rural District in the Province of Esmeraldas with an estimated total population of 150 000 that includes three towns with populations greater than 10 000 inhabitants: Quininde, La Union and La Concordia (now re-assigned to the Province of Santo Domingo following a recent plebiscite). About 70% of the cohort lives in rapidly expanding urban and peri-urban neighborhoods of these three towns, and the remainder in rural settlements. The main sources of income are derived from African palm oil and fruit cultivation, cattle, and extraction of timber. The Province is one of the poorest regions of Ecuador, with a per capita income of less than USD$2000 in 2005. The District has an ethnically mixed population of mestizos (90%), Afro-Ecuadorians (7%), and indigenous Amerindians (term used interchangeably with native) (3%) [15]. In the town of Quinindé, approximately 90% of the population have access to electricity, 60% to treated drinking water, 40% to sanitation; 60% to solid waste disposal services. In contrast, in the rural areas, 10% have access to electricity and none have access to other services.

### 2.3 Inclusion criteria and ethics approval

Formal recruitment into the cohort occurred around the time of birth. Entry criteria into the study were: 1) healthy normal baby less than 14 days old; 2) at least one stool sample collected from the mother; 3) the family had lived in the District for, at least, the last 2 years and did not plan to move out from the District over the following 3 years; 4) accessible home; and 5) maternal age of 17 years or older [15]. In the present analyses only children with complete data for the main study covariates were included. Ethics approval for the study was granted by the Ethics Committees of the Hospital Pedro Vicente Maldonado and the Universidad San Francisco de Quito, and the study is registered as an observational study (ISRCTN 41239086). The child's mother or legal guardian received both written and verbal information in Spanish about the study and provided written informed consent.

### 2.4 Measurements

First measurements of weight and height were done between birth and 2 weeks of age and then repeated periodically as close as possible to 7, 13, 24, 36 and 60 months during clinic and home visits. At each observation time, length (if less than 24 months of age) or height, in centimeters, and weight, in kilograms, measurements were taken in duplicate by trained members of a team of health professionals, and the mean of two measurements was considered as the final measurement [16]. Birth weight and length was obtained from hospital maternity records. Children were weighed without clothes or using only light underwear (without diapers) on portable electronic balances (Seca, Germany) accurate to within 100 grams. Length or height measurements were taken using in house wooden infantometers or stadiometers for children below and above 24 months of age, respectively. Z scores for weight-for-age (WAZ), height-for-age (HAZ) and weight/height-for-age (WHZ) at each observation time were calculated using WHO growth standards [17]. Additionally, to provide data on stunting and wasted prevalence, chronic malnutrition (stunting) and acute malnutrition (wasted) was defined by z scores of ≤ − 2 for HAZ and WAZ [17].

Data on socioeconomic and environmental factors were obtained using standardized questionnaires administered to the child's mother or primary care giver.

### 2.5 Statistical analyses

Individual growth trajectories for WAZ, HAZ and WHZ Z-scores were estimated from birth to 60 months of age using WHO standards and multilevel linear models. Four specifications of the multilevel models for the trajectories were used [18], [19]: (1) linear model only with a random intercept; (2) linear model with random intercept and random slope for age; (3) quadratic age model only with a random intercept; and (4) quadratic age model with random intercept and random slope for age. The choice of the best model was based on Akaike information criterion (AIC) [20]. For all outcomes, the selected best model was the multilevel quadratic age model with both random intercept and slope. After evaluation of the data dependence structure, the covariates included in the multilevel model were: number of children <15 years of age in the house, area of residence, maternal marital status and ethnicity, number of persons in house, number of natural children of mother, monthly family income and breastfeeding. Means ± SD estimated trajectories were also presented stratified by ethnicity and gender for HAZ, WAZ and WHZ. The evaluation of the final model was made through assessment of the normal distribution assumption for the errors. No outliers were detected. The methodologies for analysis of longitudinal data, such as multilevel models, can deal with missing data and still provide consistent estimates depending upon the missing data mechanism, thus avoiding the need for imputation. Multilevel models have the advantage of being applicable under relatively weak assumptions regarding the missing data mechanism and potential bias is avoided with maximum likelihood inference under any mechanism except where not random [21]. Analyzes were performed using R software, version 2.9.1 [22], and STATA, version 10; [23].

## 3 Results

**Fig. 1:**
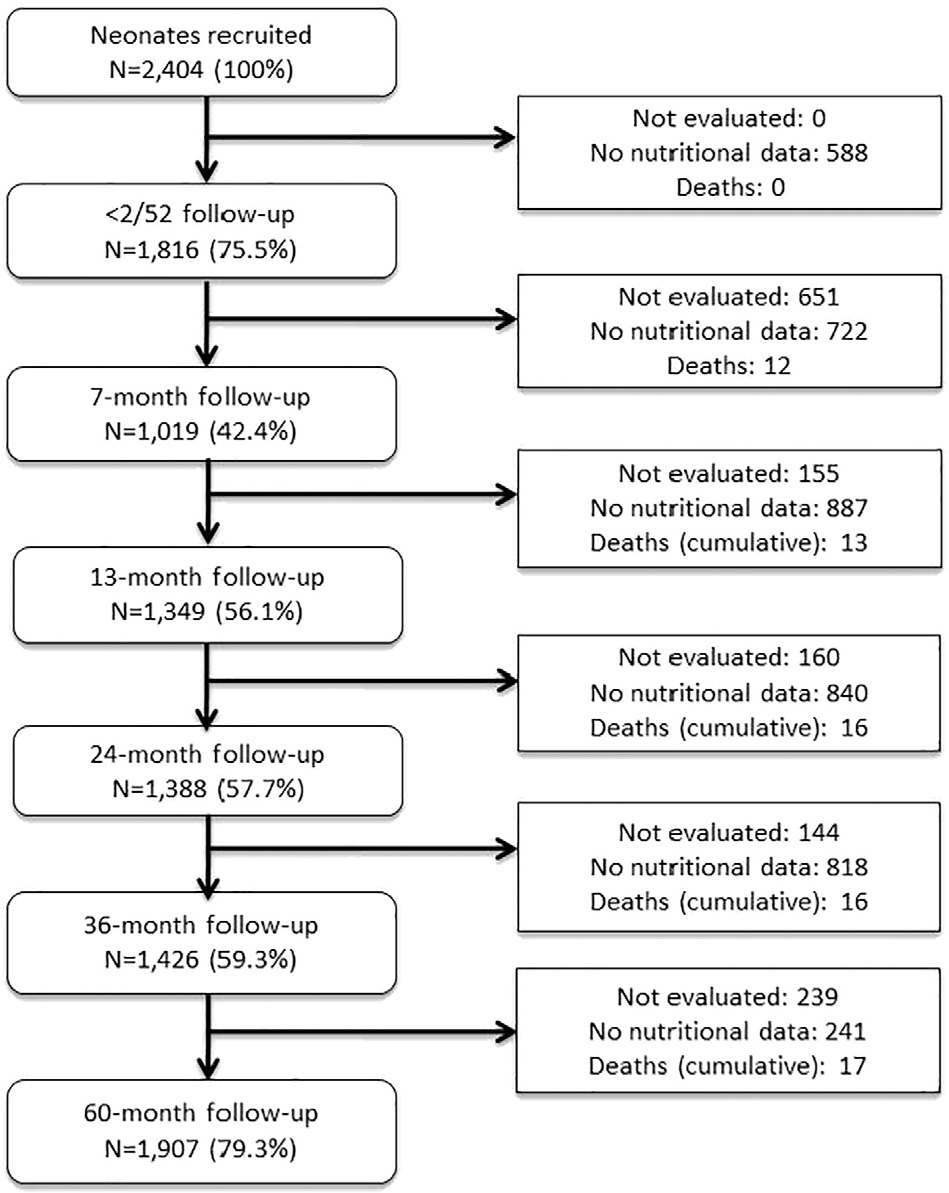
Illustration for the selection of the study population.

Of the 2404 children recruited at baseline, anthropometric data to five years of age following the initial measurement within the first 2 weeks of life were available for 1907 (79.3%) (Fig. 1). The prevalence of stunting and wasted, respectively at 24 months was 9.5% and 6.0% and at 60 months was 7.8% and 5.0%. Characteristics of study children are shown in Table 1: 25.3% of children were of Afro-Ecuadorian ethnicity, 69.7% lived in an urban or peri-urban area, approximately half lived in households with more than two children (54.1%), and a high proportion received exclusive breast milk (90.3%) for a period of greater than 3 months (47.2%).

**Table 1:** Sociodemographic and other relevant variables in study participants and mothers. Ecuador, 2005–2009

	N	%
Gender		
Male	1014	51.0
Female	973	49.0
Children ethnicity		
Afro-Ecuadorian	502	25.3
Mestizo/Native	1485	74.7
Residence		
Urban/peri-urban	1384	69.7
Rural	603	30.3
Maternal marital status		
Single, divorced or widowed	177	8.9
Married	1810	91.1
Mother no. of children		
≤2	993	50.0
>2	994	50.0
⁎Number of children <15 years of age in the household		
≤2	691	45.9
>2	815	54.1
No. of persons living in house		
≤4	587	29.5
>4	1400	70.5
⁎Breast-feeding		
No	146	9.7
Yes	1355	90.3
⁎Breast-feeding duration (months)		
zero	265	17.7
1–3	527	35.1
>3	709	47.2

N = 1987.

⁎ n least than sample.

Anthropometric characteristics of study children are shown in Table 2. Sample sizes differ by observation times because of missing data. Mean birth weight was 3.4 Kg, ranging 1.8 to 5.0 Kg. High birth weight (>4.0 Kg, 11.5%) was more frequent than low birth weight (<2.5 Kg g, 2.8%). While means weight and length/height increased with age, means weight-for age and height-for-age z scores declined with age up to 24 months after which z scores improved slightly to 60 months.

**Table 2:** Anthropometrics characteristics of cohort participants. Ecuador, 2005–2009

	N	Means (± SD)	(Min; Max)
Weight (kg)			
Birth to 15 days	1816	3.4 (0.48)	(1.8; 5.0)
7 months	1019	7.9 (1.05)	(5.4; 12.2)
13 months	1349	9.1 (1.13)	(5.8; 13.8)
24 months	1338	11.1 (1.33)	(7.2; 20.1)
36 months	1426	13.2 (1.54)	(7.9; 21.1)
60 months	1907	17.4 (2.57)	(11.2; 35.4)
Length/Height (cm)			
Birth to 15 days	1816	49.6 (2.08)	(45.0; 58.0)
7 months	1019	67.3 (2.58)	(59.0; 75.0)
13 months	1349	74.3 (2.89)	(65.0; 87.4)
24 months	1338	83.4 (3.24)	(73.0; 93.0)
36 months	1426	91.8 (3.57)	(77.6; 105.0)
60 months	1907	106.3 (4.64)	(91.0; 125.0)
HAZ (Z-Score)			
Birth to 15 days	1239	−0.30 (1.10)	(−3.92; 4.29)
7 months	989	−0.60 (1.07)	(−3.74; 3.26)
13 months	1064	−1.03 (1.11)	(−5.46; 4.31)
24 months	991	−1.16 (1.00)	(−4.94; 1.74)
36 months	1028	−1.13 (0.92)	(−4.81; 2.13)
60 months	1907	−0.76 (0.94)	(−3.88; 2.38)
WAZ (Z-Score)			
Birth to 15 days)	1546	−0.06 (0.97)	(−3.94; 3.01)
7 months	989	−0.27 (1.08)	(−3.74; 3.40)
13 months	1263	−0.58 (1.03)	(−4.00; 3.13)
24 months	1261	−0.73 (0.97)	(−4.32; 4.11)
36 months	1319	−0.68 (0.90)	(−4.99; 2.97)
60 months	1907	−0.46 (0.98)	(−3.68; 4.59)
WHZ (Z-Score)			
Birth to 15 days	1546	0.14 (1.42)	(−4.96; 4.94)
7 months	989	0.18 (1.15)	(−4.01; 4.31)
13 months	1263	−0.14 (1.05)	(−4.17; 3.50)
24 months	1261	0.01 (0.98)	(−4.16; 4.70)
36 months	1319	0.07 (0.91)	(−2.59; 4.30)
60 months	1907	0.02 (0.99)	(−4.42; 6.35)

**Fig. 2:**
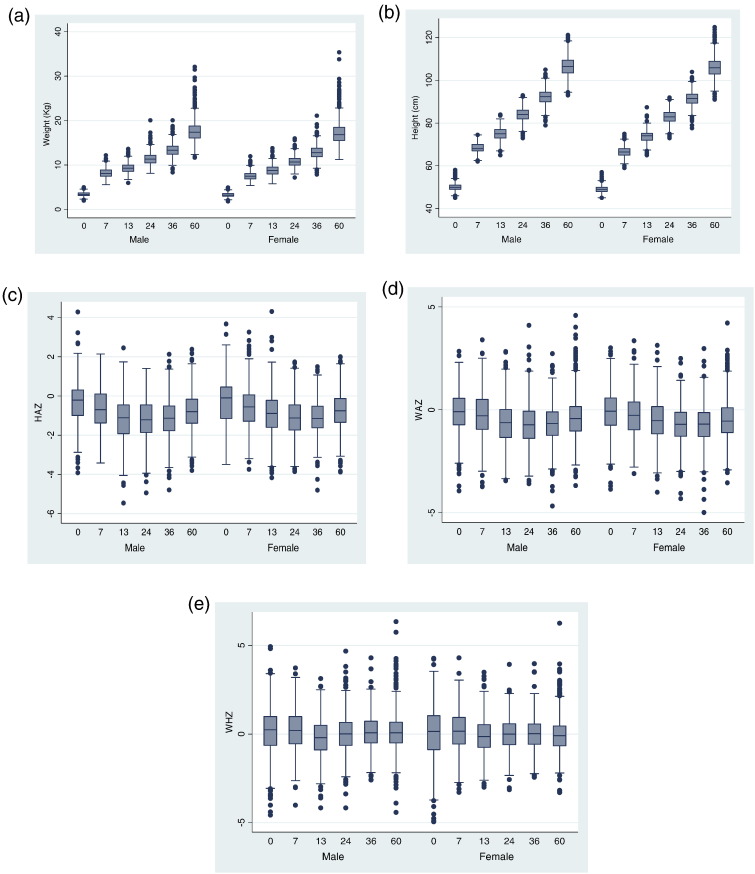
Anthropometric characteristics stratified by age and sex: (a) Weight; (b) Height; (c) HAZ; (d) WAZ; (e) WHZ. Ecuador, 2005–2009.

**Fig. 3:**
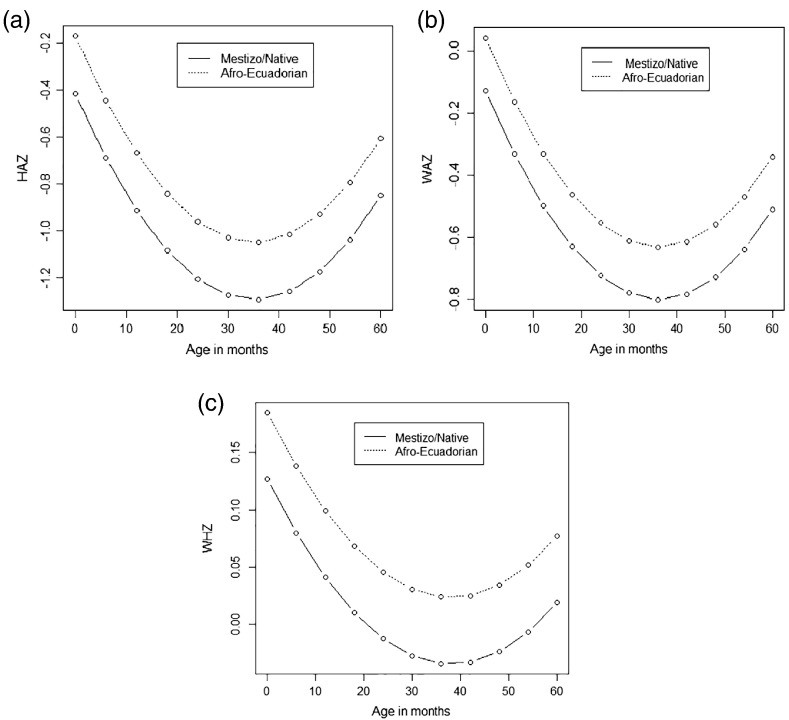
Growth trajectories* for (a) HAZ, (b) WAZ and (c) WHZ stratified by child's ethnicity**. Ecuador, 2005–2009. * Means ± SD estimated **Native = Indigenous or Amerindian children.

Fig. 2 shows anthropometric characteristics stratified by sex. Although HAZ and WAZ scores did not differ appreciably by sex (Fig. 2c and 1d), there was evidence that boys tended to heavier and longer/taller than girls at each observation time. Fig. 3 shows predicted growth trajectories for HAZ, WAZ and WHZ z scores by ethnicity using the results from the age quadratic multilevel models: mestizo/native children had greater z-score deficits throughout their trajectories for all three growth parameters compared to Afro-Ecuadorians. Afro-Ecuadorian children tended to be heavier and taller at each observation time for both sexes with better growth trajectories in both sexes.

We also fitted multilevel quadratic models for HAZ, WAZ and WHZ (Table 3, Table 4 and 5, respectively) in which relevant socioeconomic variables were included (Model 1). Breast-feeding was not statistical significantly associated with any of the 3 growth parameters and was excluded from the final analyses. Covariates with *P* > .20 were then excluded from a second model (Model 2). Among the Afro-Ecuadorian, z-scores were on average 0.25 (HAZ) and 0.17 (WAZ) higher than those observed for Mestizo/native children and this difference persisted after adjusted for all other variables in the model including heterogeneity of subjects (taken into account by random effects), which can be considered to represent unmeasured variables such as genetic predisposition, social, and environmental factors. Boys had on average 0.10 higher WHZ Z-scores than girls.

**Table 3:** Effect estimates on HAZ. Ecuador, 2005–2009

**Variables**	Model 1⁎		Model 2⁎	
	**Estimates**	**SE**	**Estimates**	**SE**
Socioeconomics				
Age	−0.05⁎⁎	0.00	−0.05⁎⁎	0.00
Age^2^	0.00⁎⁎	0.00	0.00⁎	0.00
Sex (male)	0.06	0.04	0.06	0.04
Residence (Rural)	−0.01	0.04		
Mother marital status (Single)	0.10	0.07		
Ethnicity (Mestizo/Native)	0.25⁎⁎	0.04	0.25⁎⁎	0.04
No. of persons in house (≤4)	−0.07	0.05		
No. of children (≤2)	−0.11⁎⁎	0.04	−013⁎⁎	0.04
Monthly income (US$)	0.00	0.00	0.00	0.00
Random effects				
Intercept	0.59⁎⁎	0.03	0.60⁎⁎	0.03
Age	0.00	0.00	0.00	0.00
Cov (Age, intercept)	0.00	0.00	0.00	0.00
Residual	0.43⁎⁎	0.01	0.43⁎⁎	0.01

⁎ Multilevel age quadratic models.

⁎⁎ *P* < .05.

**Table 4:** Effect estimates on WAZ. Ecuador, 2005–2009

**Variables**	Model 1⁎		Model 2⁎	
	**Estimates**	**SE**	**Estimates**	**SE**
Socioeconomics				
Age	−0.04⁎⁎	0.00	−0.04⁎⁎	0.00
Age^2^	0.00⁎⁎	0.00	0.00⁎⁎	0.00
Sex (male)	−0.01	0.04	−0.01	0.04
Residence (Rural)	−0.03	0.04		
Mother marital status (Single)	0.06	0.07		
Ethnicity (Mestizo/Native)	0.17⁎⁎	0.04	0.17⁎⁎	0.04
No. of persons in house (≤4)	−0.08	0.05		
No. of children (≤2)	−0.05	0.04		
Monthly income (US$)	0.00	0.00		
Random effects				
Intercept	0.53⁎⁎	0.03	0.53⁎⁎	0.03
Age	0.00	0.00	0.00	0.00
Cov (Age, intercept)	0.00	0.00	0.00	0.00
Residual	0.35⁎⁎	0.01	0.35⁎⁎	0.01

⁎ Multilevel age quadratic models.

⁎⁎ *P* < .05.

**Table 5:** Effect estimates on WHZ. Ecuador, 2005–2009

**Variables**	Model 1⁎		Model 2⁎	
	**Estimates**	**SE**	**Estimates**	**SE**
Socioeconomics				
Age	−0.01⁎⁎	0.00	−0.01⁎⁎	0.00
Age^2^	0.00⁎⁎	0.00	0.00⁎⁎	0.00
Sex (male)	−0.10⁎⁎	0.04	−0.10⁎⁎	0.04
Residence (Rural)	−0.03	0.04		
Mother marital status (Single)	−0.01	0.07		
Ethnicity (Mestizo/Native)	0.06⁎⁎	0.04		
No. of persons in house (≤4)	−0.05	0.05		
No. of children (≤2)	−0.01	0.07		
Monthly income (US$)	0.00	0.00	0.00⁎⁎	0.00
Random effects				
Intercept	0.52⁎⁎	0.03	0.52⁎⁎	0.03
Age	0.00	0.00	0.00	0.00
Cov (Age, intercept)	0.00	0.00	0.00	0.00
Residual	0.71⁎⁎	0.02	0.71⁎⁎	0.02

⁎ Multilevel age quadratic models.

⁎⁎ *P* < .05.

## 4 Discussion

Ecuadorian boys were heavier and had a greater predicted mean length at birth than girls while Afro-Ecuadorian babies, as hypothesized, were heavier and longer than mestizo or Amerindian children independent of sex and had better growth trajectories for height-for-age (HAZ) and weight-for-age (WAZ) during the first five years of life. The latter observation was independent of sex and socioeconomic factors with the exception of number of children living in the household that was associated with greater deficits in HAZ. These findings contribute to our understanding of anthropometric trajectories and ethnicity in Latin America.

The stunting rates observed in our study were consistent with the findings of previous studies in Latin America [24] in which the countries with the highest rates of stunting were those with a greater presence of indigenous populations. However, it has been also noted in studies conducted in Brazil, Mexico, Chile and Venezuela that child growth deficits are independent of ethnicity and are influenced by improvements in health and the introduction of cash transfer programs among vulnerable populations [25], [26], [27]. Such studies indicate that stunting was not influenced by ethnicity but by low socioeconomic status and exposure to an unfavorable environment. Longitudinal growth of children is considered to be a good indicator of the quality of the environment in which they have lived and has been used as a global indicator of the quality of life [25], [26].

Studies of heritability have indicated genetics may account for up to 60% of adult height [28] However, as economic conditions improve with development in Latin America accompanied by improvements in education and living environment, an increasing proportion of the population are likely to achieve their maximum genetic potential in terms of height. However, development in Latin America has been accompanied by increasing levels of inequality, and under such circumstances, socioeconomic differences within populations may become an increasingly important determinant of variability in height [22], [23]. Further, at the population level the prevalence of chronic malnutrition is often used as an indicator of economic development and the overall health of a society, with child growth being determined by a dynamic interaction of genetic and environmental factors [24]. An enabling environment with good access to healthy food, hygiene, health care, affection, among other factors, provides the necessary conditions for children to develop their growth and height potential regardless of ethnicity. Mothers and children without family support or who live in poor rural areas represent a vulnerable group for malnutrition. However, neither family support nor vulnerable environment was related to child growth in our study. Chronic malnutrition in rural areas of Ecuador was estimated previously to be 32.9% compared to 19.7% in urban areas [12]. In our study population, children born in families with single mothers were infrequent (8.9%) and it is possible that in the study area, differences between poor rural, urban or peri-urban environments, may not be sufficiently great to allow us to detect differences in malnutrition. Another potential explanation is the positive effect of Ecuador's cash transfer program (‘bono solidario’) on the growth of rural children as has been observed in other studies [31], thus reducing differences by area of residence.

Recent population based studies in Ecuador [10], [12], [25] showed a decline in the rate of stunting from 40.2% in 1986 to 25.3% in 2012, with a rate of 17.7% in Afro-Ecuadorian children aged up to 5 years. In this study we observed a lower prevalence of stunting in 7.8% in children to 5 years of age but with a different set of reference standards. Stein and colleagues (2010) analyzed longitudinal data from five birth cohorts in low- and middle-income countries, showing growth restriction from birth to 2 years with a modest recovery in mid-childhood. Such a recovery was also observed in our study after 30 months of age.

Several studies have shown a clear relationship between birth weight and later childhood growth - children with low birth weight having accelerated growth during the first two years of life while heavier children had slower growth [26]. The low prevalence of low birth weight observed in this study (2.8%) is much lower than estimated in a previous survey in Ecuador in which 11% of children had low birth weight [13]. However, these data may be difficult to compare because the initial measurement made in our study was done between birth and 14 days of age and the cohort was of healthy term newborns. Birth weight is a good indicator of factors that determine intrauterine growth and, in turn, is a predictor of risk for adiposity and metabolic syndrome in later life within the concept of metabolic programming and body composition [27], [28]. In a recent study of term newborns, predominantly indigenous, in nine northern and central Ecuadorian provinces, 16.9% of newborns had low birth weight. The same study showed that Afro-Ecuadorian children (4.5% of the total population) were fatter at birth than mestizo children and only 1.8% had low birth weight [29]. In our population, therefore, in which a significant proportion were of African ancestry, the prevalence of low birth weight was lower than the latest national estimate (8%) [33].

Previous studies have shown differences in height and weight between Afro-Ecuadorian and mestizo women, and that infants of Afro-Ecuadorian mothers were heavier [29], [30]: newborns of Afro-Ecuadorian mothers weighed 5% (150 g) more than those of mestizos, a difference that remained statistically significant after controlling for confounding [32]. Previous studies have shown that the risk of obesity in adulthood in increased not only among children with low birth weight but also among those with high birth weight [34], [35], [36]. High birth weight (ie >4 kg) in this study was more frequent than low birth weight (11.5% vs. 2.8%, respectively).

Our data show that children aged around 36 months were at greater risk of stunting particularly non Afro-Ecuadorian children. Short period of breastfeeding, inadequate introduction of complementary foods and low coverage and quality of public health interventions may explain the greater vulnerability at this age [11]. Nevertheless, we did not observe any association of growth parameters with breastfeeding in this population.

The findings from this study should be interpreted in light of its limitations and strengths. A major strength of this study is the availability of six direct measurements of growth parameters taken prospectively between birth and 5 years of age by a trained team using standardized methods. However, first measurements of weight and height were done between birth and 2 weeks of age. Residual confounding is a potential weakness given that the study is observational.

Many lower and middle-income countries, such as Ecuador, have seen alterations in feeding practices and energy expenditure in children over the last decade as a consequence of changes in economic, social, demographic and health structures. These changes are known as the ‘nutrition transition’ and provide an opportunity to investigate the role of nutrition in the causation of chronic diseases such as type-2 diabetes that has reached epidemic levels in many Latin American countries. The main consequence of the nutrition transition has been a predominance of diet-related chronic diseases and increases in weight. Further studies on growth in early life will be important, especially in developing countries, which are experiencing such a transition and which is likely to be associated with increases in the occurrence of overweight/obesity, particularly among the socially vulnerable.
